# A Real-World Experience of Azathioprine Versus First-Line Disease-Modifying Therapy in Relapsing-Remitting Multiple Sclerosis—A Prospective Cohort Study

**DOI:** 10.3390/brainsci13091249

**Published:** 2023-08-27

**Authors:** Arpit Agrawal, M. V. Padma Srivastava, Rohit Bhatia, Vinay Goyal, Mamta Bhushan Singh, Venugopalan Y. Vishnu, Anuj Prabhakar

**Affiliations:** 1 Department of Neurology, All India Institute of Medical Sciences (AIIMS), New Delhi 110029, India; arpdoc@gmail.com (A.A.); rohitbhatia71@yahoo.com (R.B.); drvinaygoyal@gmail.com (V.G.); mbsneuro@gmail.com (M.B.S.); vishnuvy16@yahoo.com (V.Y.V.); 2 Department of Neuroradiology, All India Institute of Medical Sciences (AIIMS), New Delhi 110029, India; dranujprabhakar@gmail.com

**Keywords:** azathioprine, relapsing-remitting multiple sclerosis, first-line disease-modifying therapy, dimethyl fumarate, interferon beta-1a, teriflunomide

## Abstract

Azathioprine (AZA) has demonstrated efficacy in multiple randomized control trials (RCTs) for Relapsing-Remitting Multiple Sclerosis (RRMS). However, we still need comparative real-world data with other first-line disease-modifying therapies (DMTs). We aimed to assess AZA’s effectiveness regarding relapses, disability progression, time to the first relapse, magnetic resonance imaging (MRI) activity, and safety compared with other approved first-line DMTs in an Indian population in a real-world setting. We conducted a single-center prospective study of treatment-naive RRMS patients between 2017 and 2019. We evaluated the effects of AZA and other approved DMTs on clinical and radiological measures. Among 192 eligible patients (F:M ratio 2.84:1), 68 patients (35.4%) were on AZA, 68 patients (35.4%) were on dimethyl fumarate (DMF), 32 patients (16.7%) on interferon (IFN beta-1a), and 16 patients (8.3%) on teriflunomide (TFL). Four treatment groups were comparable: AZA v/s DMF v/s TFL v/s IFN beta-1a. In primary outcomes, there was no significant difference between the groups in terms of change in the Expanded Disability Status Scale (EDSS) score at three months (*p*-value = 0.169), six months (*p*-value = 0.303), 12 months (*p*-value = 0.082), and 24 months (*p*-value = 0.639), the number of relapses (*p*-value = 0.229), and time to the first relapse (*p*-value > 0.05 in all groups). In the secondary outcome, there was no significant difference between the treatment groups on serial MRI parameters used according to “Magnetic Resonance Imaging in Multiple Sclerosis” (MAGNIMS) 2016 criteria (*p*-value > 0.05). In safety outcomes, leukopenia was significantly more common in the AZA group (*p*-value = 0.025), flu-like symptoms (*p*-value = 0.0001), and injection site reactions (*p*-value = 0.035) were significantly more common in the IFN beta-1a group. Our study suggests AZA is as effective as other approved DMTs and a good alternative as a first-line treatment for multiple sclerosis’s clinical and radiological activity in real-world settings on short follow-up. Based on these results, more randomized controlled trials of AZA v/s DMF or other DMTs are needed for more robust outcomes.

## 1. Introduction

Researchers continuously work to find safer and more efficacious treatments for Multiple Sclerosis, hoping to stop relapses and disability progression. Disease-modifying therapies (DMTs) reduce the occurrence of relapses, slow neurological disability, and prevent the decline of patients’ quality of life [1,2]. Azathioprine (AZA), a nitro-imidazole substituted form of 6-mercaptopurine, has received considerable attention as a potential therapeutic agent in MS [3,4,5]. It has limited toxicity, is inexpensive, and is easy to administer and monitor [3,4]. In comparison, up to 45% of patients on newer biologicals may experience adverse events [6].

Moreover, the safety profile of DMTs may differ in real-world populations compared with subjects in clinical trials [4,7]. A beneficial effect of AZA on disability progression and the relapse rate was also found [8,9,10,11]. Treatment costs play a significant role in health care delivery in developing countries such as India, where most patients do not have health insurance. Since the disease activity is supposed to be at its maximum at the outset of illness and the patient may develop disabilities that may or may not fully improve subsequently [12,13,14,15] there is a window of opportunity for treatment that should not be lost.

AZA has been used to treat RRMS based on placebo-controlled RCTs [3,4,16]. However, efficacy was usually considered marginal [3], and after approval of β-interferons, AZA fell out of repute as first-line therapy. Lack of MRI evaluation, methodological weaknesses, and the low power of the trials may have caused a perception of the poor efficacy of AZA. In contrast, consistently efficacious and safe interferon trials in MS have made interferons a drug of choice for this indication. However, meta-analyses, comparative RCTs, and MRI results suggest a similar effect size of AZA in RRMS [3,4,8,9,10,11,16,17,18]. A Cochrane review on AZT for multiple sclerosis in 2007 described AZT as the most widely used immunosuppressive agent for RRMS [4]. However, a Cochrane review in 2017 labeled AZT as “off-label” [19]. After that, AZT has yet to attract researchers’ interest, so no further RCTs were conducted. After reviewing the literature, we conclude that AZT was underutilized despite the initial evidence, probably due to a lack of recent evidence. Considering the recent trends favoring oral treatments for MS, a revisit of AZT’s role compared with current treatments can be worthwhile. This study aims to revisit the role of AZA in the current treatment of RRMS and compare it to other approved DMTs.

## 2. Materials and Methods

### 2.1. Study Design

We conducted a prospective observational study on 192 patients at a single center. It compared the efficacy and safety of AZT v/s other approved first-line DMTs in treating RRMS in terms of the number of relapses and EDSS (Expanded Disability Status Scale) score [20], allowing for a better assessment of the effects of a medication administered in a larger patient population. We treated all the patients following the approved label instructions and the expected standards of good clinical practice. The AIIMS (All India Institute of Medical Sciences, New Delhi, India) Institute Ethics Committee approved the study protocol.

### 2.2. Study Population

The study population consisted of adult patients (≥18 to ≤55 years) diagnosed with active Relapsing-Remitting Multiple Sclerosis (RRMS) according to McDonald criteria 2010 [21] (see Appendix A and Appendix B for detailed inclusion and exclusion criteria and detailed McDonald criteria 2010, respectively). We screened 256 consecutive RRMS patients in the MS clinic for eligibility, out of which we included 192 prospective treatment-naïve RRMS patients in the study. The patient or the caregiver gave written informed consent for participation in the study.

### 2.3. Data Acquisition and Definitions

We followed up with eligible patients for six months to two years. We excluded patients with less than six-month follow-up from the study due to the short time interval for clinical relapse. The treating physician decided to initiate therapy based on the nature of DMT and its switching. We gave symptomatic treatment for relapses through intravenous methylprednisolone 1g over 3–5 days or oral steroids. The examining neurologist (MVP, RB, VVY) oversaw the overall medical management of patients, including drug prescription and self-administration instructions, scheduled (quarterly) and unscheduled (i.e., at the onset of new symptoms or complications) follow-up visits. They recorded symptoms, blood test results, clinical adverse events and their management, and any treatment decision, including discontinuation.

The other neurologist (AA) was responsible for the neurological examination and EDSS scoring at scheduled and unscheduled visits to confirm relapses. All the assessments were completed by the same neurologist (AA). These included the onset of new neurological symptom(s) or worsening of pre-existing ones from MS, determining the worsening of at least one point in one or more functional systems (FS) or at least 0.5 EDSS points [20]. We considered a new symptom a recent relapse if it lasted at least 48 h with no fever and was reported at least 30 days after a previous relapse. Participants were followed up regularly at 3, 6, 12, and 24 months during their routine follow-up OPD (Outpatient Department) visits. Per current guidelines, the treating physician gave the patients a basket of therapeutic options with a detailed discussion of therapeutic benefits and potential side effects. We decided on the therapeutic drug after a one-on-one discussion between the patient or caregiver and the treating physician. Most of the patients chose oral medications over the subcutaneous route. We offered the option of Azathioprine to patients who could not afford approved DMT. The dose of AZT was 2–3 mg/kg; DMF was used at a dose of 120 mg twice a day for the first seven days, followed by 240 mg twice a day; teriflunomide was used at a dose of 7 mg/day for 14 days, followed by 14 mg/day; and interferon beta-1a (Avonex) was used at a dose of 30 micrograms weekly. We advised the women in the childbearing age group to use an effective contraception method (see Appendix A for details about contraception methods). We prescribed only IFN beta-1a to women who were planning a pregnancy. We monitored all the patients according to the drug used and its respective side effect profile. All the participants (or their authorized representatives) gave written informed consent.

The brain MR imaging protocol included 3D T1-weighted, 3D T2-FLAIR, 3D T2-weighted, post-single-dose gadolinium-enhanced T1-weighted sequences, and a DWI sequence with a non-gapped section thickness of <3 mm and a DWI sequence (<5-mm section thickness). The spinal cord MR imaging protocol included sagittal T1-weighted and proton attenuation, STIR or phase-sensitive inversion recovery, axial T2- or T2*-weighted imaging through suspicious lesions, and in some cases, postcontrast gadolinium-enhanced T1-weighted imaging. We did all MR imaging on 3 Tesla MRI machines (PHILIPS, Ingenia, made in Best, Netherlands) with adequate SNR and spatial resolution (in-section pixel resolution of ≤1 × 1 mm). Reconstruction (interpolation) was achieved at 0.5 mm. Routine brain MR imaging was considered every six months to two years for all patients with relapsing MS or when there was a relapse. As this is a real-world study, not all patients underwent routine imaging at 1-year or 2-year intervals, and serial imaging of only 49 patients was available for analysis. MRI outcomes were scored according to the pre-set format, which a neuroradiologist (AP) confirmed. A contextual template for MS follow-up was used based on MAGNIMS 2016 MRI criteria [22,23,24]. For the reason that the template focuses on items pertinent to the clinical indication, reporting can be accomplished rapidly, permitting quick tabbing through the fields.

We considered all DMTs, approved or off-label, that are currently under marketing authorization or investigation for people with a first clinical attack. We believed all agents used or under investigation for RRMS could be given to people with a first attack complying with the 2010 McDonald criteria [21].

The study was conducted following the Declaration of Helsinki and the principles of Good Clinical Practice. This study was based on GRACE (Good Research for Comparative Effectiveness) principles, so it was a prospective study to generate high-level evidence for observational studies.

Primary outcome measures were a comparison of the change in mean EDSS score, the number of relapses, and time to first relapse of patients on Azathioprine v/s disease-modifying approved therapy. The secondary outcome measure was a comparison of changes in MRI parameters in New T2/FLAIR lesions, an increase in lesion size, new enhancing lesions, new T1 hypointensity, parenchymal atrophy, callosal atrophy, and an increase in overall disease burden.

### 2.4. Statistical Analysis

We proposed to recruit a total of 200 patients over a two-year period based on the number of patients attending the MS clinic. Data were analyzed by SPSS (Statistical Package for the Social Sciences) version 14 and presented as mean (SD)/Median (minimum-maximum) and frequency percentage. We used the Kruskal-Wallis test to compare the change in EDSS score among the 4 groups (i.e., AZA, DMF, IFN, and TFL) as this test compares continuous variables among the groups, followed by multiple comparisons using the Dunn test with Bonferroni correction. We used the chi-square and Fischer’s exact tests to compare MRI outcomes among various groups as they dealt with categorical variables. We carried out Kaplan-Meier analysis to see the relapse pattern among the four treatment groups, and we compared the relapse pattern by log-rank test. Univariate Cox’s regression analysis calculated the Hazard ratio with a 95% Confidence Interval for time to the first relapse. A *p*-value < 0.05 was considered statistically significant.

## 3. Results

### 3.1. Descriptive Analysis

**Figure 1:**
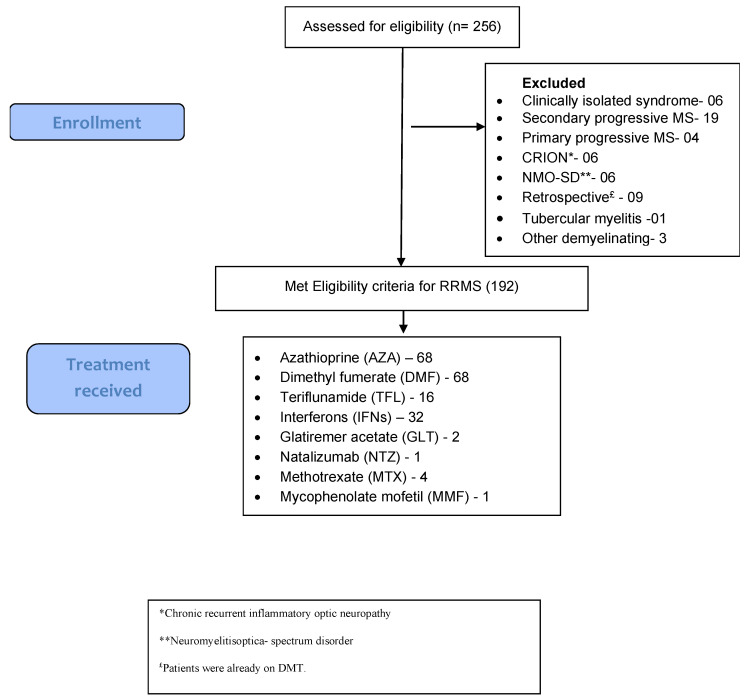
Overview of the study.

We screened 256 patients for the study attending the MS clinic; 192 (50 male, 142 female) qualified for the study (see Figure 1). Sixty-eight patients (35.4%) were each on AZA and DMF, respectively; 32 patients (16.7%) on interferon beta-1a; 16 patients (8.3%) on TFL; two patients (1%) on GLT; four patients (2.1%) on MTX; one patient (0.5%) each on NTZ and MMF, respectively (see Table 1). No dropouts were reported in any of the groups. Four patients were on MTX because they could not afford AZA. One patient was started directly on natalizumab as she already had four relapses before the presentation. Clinic-radiological data suggested Highly Aggressive Multiple Sclerosis. One patient was on MMF at the treating physician’s discretion. We determined patient compliance through oral interviews. Patient compliance was good. The patients were available for follow-up at 3, 6, 12, and 24 months. We did not include the patients with follow-ups of less than six months. Twenty-two patients had a follow-up of 6 months, 71 patients for 12 months, and 99 patients had a follow-up of 24 months. The F:M ratio was 2.84:1. The difference in gender between the groups was insignificant. The difference between the groups for other baseline characteristics was also insignificant (see Table 2).

### 3.2. Comparison of Expanded Disability Status Scale (EDSS) before and after Treatment

We used the Kruskal-Wallis test to compare the change in EDSS between the two groups, AZA, DMF, TFL, and IFN beta-1a. We could not compare this parameter to other groups due to the fewer patients. Changes in EDSS at three months (*p*-value = 0.303), six months (*p*-value = 0.132), 12 months (*p*-value = 0.082), and 24 months (*p*-value = 0.639) were not significant among all four groups (see Table 3).

### 3.3. The Number of Relapses in Different Treatment Groups

We compared the hazard ratio for the number of relapses between the four groups. Although the various groups had different absolute numbers of relapses, the difference was not statistically significant (*p*-value = 0.229) (see Table 4).

### 3.4. Time to First Relapse

**Figure 2:**
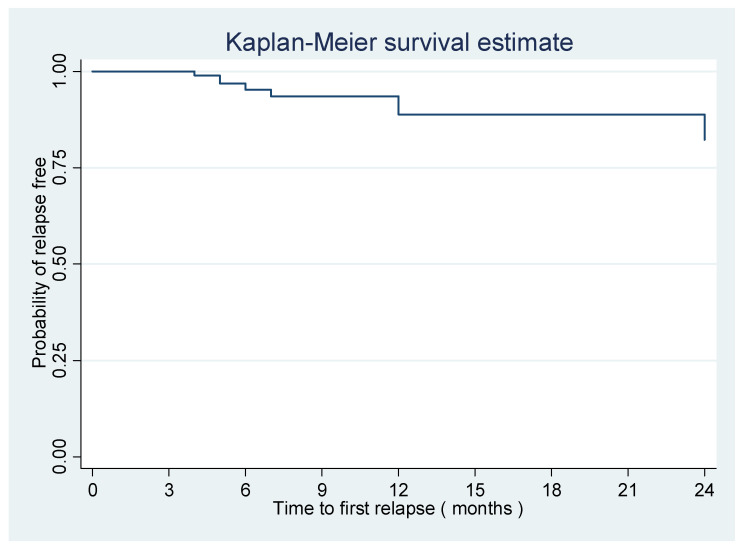
Kaplan Meier curve showing the overall probability of relapse-free survival.

**Figure 3:**
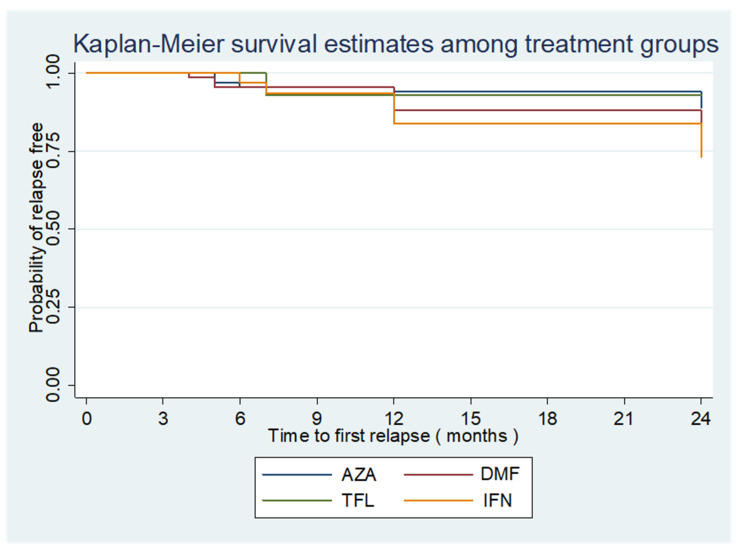
Kaplan Meier curve showing the probability of relapse-free survival in different treatment groups. AZA = Azathioprine, DMF = Dimethyl fumarate, TFN = Teriflunomide, IFN = Interferon beta-1a.

Using the log-rank and Cox proportional hazards models, we compared the time to the first relapse between the groups. The four treatment groups had no significant difference (Table 5). Figure 2 and Figure 3 show the Kaplan-Meier curves of the time to first relapse using the log-rank test and Cox’s proportional hazards models. Figure 2 shows the overall time to first relapse, and Figure 3 shows the time to first relapse between different groups. The groups did not have significant differences (*p* value > 0.05).

### 3.5. MRI Outcomes

MRI outcomes were scored according to a pre-set format by the author (AA), which was reconfirmed by an expert neuroradiologist (AP). A contextual template for multiple sclerosis follow-up was used based on the MAGNIMS 2016 MRI criteria [24]. Serial imaging of 49 patients was available for analysis. Of these 49 patients, 15 were on AZA, 16 on DMF, 11 on IFN beta-1a, and two on TFL. We used Chi-square and Fisher’s exact test; there was no difference between any of the groups in terms of New T2/FLAIR lesions (*p*-value = 0.137), Increase in size of the lesion (*p*-value = 0.245), New Enhancing lesions (*p*-value = 0.132), Number of T1 hypointensities (*p*-value = 0.628), Parenchymal atrophy (*p*-value = 0.703), and Callosal atrophy (*p*-value = 1.00) (see Table 6).

### 3.6. Safety Outcomes

Most of the patients tolerated the treatment well. We discontinued AZA temporarily for one patient due to leukopenia, and one patient was shifted to DMF from IFN beta-1a due to injection site pain (See Table 7). However, he had no relapse or disability progression on IFN beta-1a. Three patients in the AZA group and one patient in the TFL group had transaminitis, but the *p*-value was not statistically significant (*p*-value = 0.132) and did not require drug discontinuation. We calculated the frequency of all adverse effects using the chi-square test and Fisher’s exact test, which showed no significant difference except that leukopenia was significantly more common in the AZA group (*p*-value = 0.025). Flu-like symptoms (*p*-value = 0.0001) and injection site reactions (*p*-value = 0.035) were significantly more common in the IFN beta-1a group, and the latter two were reported in the IFN beta-1a group only. We did not report any unknown adverse events.

## 4. Discussion

RCTs assess DMT’s short-term efficacy and safety in a determined patient population. Real-world evidence (RWE) can provide long-term evidence on various endpoints, such as effectiveness, safety, patient-reported outcomes (PROs), and physician preference.

In this study, we performed a head-to-head comparison of AZA v/s other first-line DMTs. There was no significant difference in the efficacy of AZA compared with other Level 1 DMTs, i.e., DMF, TFL, or IFN beta-1a, in terms of change in mean EDSS scores, number of relapses, and the time to the first relapse. There was no difference in MRI outcomes regarding New T2/FLAIR lesions, an increase in lesion size, new enhancing lesions, new T1 hypointensity, or parenchymal and callosal atrophy (*p*-value > 0.05). Our study found AZA to be as efficacious as other first-line DMTs, considering the above parameters. However, the superiority of AZA was not detected. Ours is the first study with a head-to-head comparison of the three oral first-line DMTs: AZA vs. DMF vs. TFL [25].

The incidence of leukopenia was significantly higher in the AZA group, and transaminitis was seen only in the AZA and TFL groups. We anticipated these adverse effects and, through monitoring and dosage adjustment, they were easily managed. Hepatic dysfunction, accompanied by nausea and vomiting, is a relatively frequent side effect of AZA. However, these symptoms can also occur with TFL or even iv methylprednisolone [26,27]. These results are consistent with the Cochrane review by Casetta et al. [4] and emphasize that AZA is a safe alternative with proper monitoring. However, cumulative doses of 600 g of AZA should not be exceeded in relation to a possible increased risk of malignancy [4,28,29]. Immunosuppression with AZA increases the risk of malignancy in humans (FDA 2014).

An RCT [30] compared the efficacy of AZA vs. IFN beta-1a in terms of the mean number of relapses and the change in the mean EDSS score and showed the superior efficacy of AZA. Another RCT compared the effectiveness of AZA vs. IFN beta in terms of annualized relapse rate ratio, time to first relapse, and MRI outcomes and showed non-inferiority of AZA as compared with IFN beta.

The present study strengthens previous results of AZA vs. Placebo or AZA vs. IFN beta-1a and expands previously available data [31]. In our research, we took MRI parameters according to standard criteria designed for Multiple Sclerosis [24]; hence, MRI outcomes were more robust compared with previous studies on AZA efficacy. The previous MRI studies were informative for supporting the hypothesis of AZA efficacy on brain lesions but were not aimed at assessing clinical outcomes [32].

An RCT regarding this clinical question was not feasible in our setting due to a lack of resources. However, this prospective observational study has its distinct advantages:

1. Cost-effective as compared with an RCT.
2. Conducted in a real-life situation, as compared with a controlled environment in an RCT.
3. Practical considerations such as a clinician and patient’s preferences for DMTs, how and when they switch them, cost concerns, and determining adverse effects and long-term outcomes more thoroughly.

In an RCT by Etemadifar et al. [30] comparing the efficacy of IFN beta products and AZA among 94 treatment-naive patients for 12 months, the mean number of relapses was lower in the AZA group than in the IFN beta-1a group (0.28 vs. 0.64, *p*-value < 0.05). After 12 months, 57.4% of patients receiving IFN beta products remained relapse-free, compared with 76.6% of those given AZA. EDSS decreased by 0.30 units in IFN beta-1a-treated patients (*p* < 0.05) and 0.46 units in AZA-treated patients (*p* < 0.001).

Another RCT by Massacesi et al. [25] compared AZA v/s IFN beta-1a in 150 RRMS patients and showed non-inferiority of AZA in both clinical and MRI outcomes. In a meta-analysis by Messori et al. [31], the indirect comparison of AZA vs. IFNs showed a RR of 0.88 (95% CI: 0.78 to 1.08) for the relapse rate at 24 months. In the rankogram, placebo (as expected) consistently ranked worst; Azathioprine ranked best in nearly all simulations, while IFNs generally ranked second [31].

One of the most prominent strengths of the study is that it is a pragmatic, low-cost, real-life study of AZA and other DMTs in a resource-poor setting. Secondly, it is a direct head-to-head comparison of the drug AZA, which has been there for many decades and is still widely used in our country for RRMS, with approved DMTs in the current scenario, including MRI outcomes.

One of the main limitations of our study was the small sample size. The number of patients in the GLT, NTZ, MTX, and MMF groups was too small for analysis. Additionally, due to the smaller number of patients in the TFL and IFN groups, the results should be interpreted with caution. AZA was offered to patients with poor financial status or according to the treating physician’s choice. Moreover, women planning pregnancy were offered only the choice of IFN beta-1a since that is the only approved DMT during pregnancy; this may have introduced a selection bias in our study. However, the results were consistent with prior studies comparing AZA and IFN beta-1a. Another limitation of the study is that fewer follow-up MRIs were available for comparison, which may be a source of bias. Many patients were on treatment for only six months, so a repeat scan was unnecessary. Some patients were doing very well clinically, so the treating physician decided that a repeat scan was unnecessary and unlikely to change management. Many patients were advised to have an MRI scan but could not afford one as most of the expenditure is out of pocket in our setting.

## 5. Conclusions

Based on the benefits obtained with AZA, its low toxicity, ease of administration, and low cost, this drug is still helpful in treating RRMS. The encouraging results obtained in this study will pave the way for further studies, including a large-scale, probably blinded RCT with a more extended follow-up period. The use of AZA may help reduce the cost of treatment for RRMS. In conclusion, this comparative study of AZA and approved DMTs suggests they are equally efficacious. These results can help recommend and select DMTs for RRMS. The results of this study can be relevant for clinical practice, especially in developing nations where cost and availability can limit treatment, supporting AZA as a rational and effective alternative to approved first-line DMTs in RRMS, particularly considering the convenience of oral administration and the cost.

## Figures and Tables

**Table 1:** Number of patients in different treatment groups.

S. No	Treatment Groups	No. of Patients (*n* = 192)	Percentage (%)
1.	Azathioprine (AZA)	68	35.4%
2.	Dimethyl fumarate (DMF)	68	35.4%
3.	Teriflunomide (TFL)	16	8.3%
4.	Interferons (IFN)	32	16.7%
5.	Glatiramer acetate (GLT)	2	1.0%
6.	Natalizumab (NTZ)	1	0.5%
7.	Methotrexate (MTX)	4	2.1%
8.	Mycophenolate mofetil (MMF)	1	0.5%

**Table 2:** Baseline characteristics.

Characteristics	Treatment Group (*n* = 184)				*p*-Value
Drugs	AZA (*n* = 68)	DMF (*n* = 68)	TFL (*n* = 16)	IFN (*n* = 32)	
Mean Age in Years (SD)	29.3 (6.33)	29.3 (7.77)	27.7 (5.73)	32 (8.77)	0.216
Male Sex-number (%)	20(29.40)	11(16.10)	5 (31.20)	10 (31.20)	0.176
Female-number (%)	48 (70.59)	57 (83.8)	11 (68.75)	22 (68.75)	
Mean baseline EDSS score (SD)	1.31 (1.57)	0.73 (1.14)	1.03 (1.27)	1.14 (1.49)	0.169
Median baseline EDSS score	1	0	0	1	-
Mean no. of relapses before DMT (SD)	2.51(0.74)	2.37 (1.12)	1.93 (0.99)	2.72 (0.89)	0.132

**Table 3:** Change in EDSS score at 3 months, 6 months, 12 months, and 24 months between different treatment groups.

Drugs		EDSS at Baseline	EDSS at 3 Months	EDSS at 6 Months	EDSS at 12 Months	EDSS at 24 Months
AZA	N	68	68	68	65	41
-	Mean (SD)	1.31 (1.57)	1.17 (1.48)	1.08 (1.47)	1.08 (1.58)	0.83 (1.38)
DMF	N	68	68	68	54	26
-	Mean (SD)	0.73 (1.14)	0.65 (1.21)	0.63 (1.21)	0.60 (1.27)	0.42 (0.87)
TFL	N	16	16	16	14	8
-	Mean (SD)	1.03 (1.27)	0.78 (1.19)	0.78 (1.19)	0.75 (1.22)	0.375 (0.74)
IFN	N	32	32	32	30	25
-	Mean (SD)	1.14 (1.49)	1.11 (1.49)	1.01 (1.34)	1.13 (1.42)	0.94 (1.08)
*p*-value	-	0.169	0.303	0.132	0.082	0.639

**Table 4:** Number of relapses in different treatment groups: Hazard ratio for relapse between different treatment groups compared with Azathioprine.

Drugs as Compared with Azathioprine	Total Patients	Number of Relapses	*p*-Value	Hazard Ratio	95% Confidence Interval (95% CI)
AZA	68	6	0.229	1	-
DMF	68	9		1.88	(0.66, 5.28)
TFL	16	1		0.73	(0.09, 6.05)
IFN	32	8		2.58	(0.89, 7.44)

**Table 5:** Survival function between the different groups at 6, 12, and 18 months showing the chance of a relapse v/s patients at risk of relapse.

Drug	Time in Months	No Patients at Risk	No Events (Relapses)	Survival Function	Standard Error	95% Confidence Interval (95% CI)
AZA	6	66	3	0.956	0.025	(0.869, 0.985)
-	12	62	1	0.940	0.029	(0.849, 0.977)
-	18	62	0	0.940	0.029	(0.849, 0.977)
DMF	6	65	3	0.956	0.025	(0.869, 0.985)
-	12	51	4	0.881	0.043	(0.764, 0.942)
-	18	51	0	0.881	0.043	(0.764, 0.942)
TFL	6	16	0	1.000	-	-
-	12	13	1	0.928	0.069	(0.591, 0.989)
-	18	13	0	0.928	0.069	(0.591, 0.989)
IFN	6	32	1	0.968	0.030	(0.798, 0.995)
-	12	29	4	0.836	0.066	(0.657, 0.929)
-	18	29	0	0.836	0.066	(0.657, 0.929)

**Table 6:** MRI Outcomes among different treatment groups.

MRI Parameters-Number (%)	Treatment Groups (*n* = 49)				*p*-Value
-	AZA (*n* = 15)	DMF (*n* = 16)	TFL (*n* = 2)	IFN (*n* = 11)	
New T2/FLAIR lesions	13 (86.67)	13 (81.25)	0	6 (54.55)	0.137
Increase in lesion size	6 (40)	8 (50)	0	2 (18.18)	0.245
New-enhancing lesions	7 (36.84)	1 (5.88)	0	3 (27.27)	0.132
T1-hypointensity	5 (26.32)	6 (35.29)	1 (50)	5 (45.45)	0.628
Parenchymal atrophy	2 (13.33)	2 (11.76)	0	3 (27.27)	0.703
Callosal atrophy	1 (6.67)	2 (11.76)	0	1 (9.09)	1.000

**Table 7:** Adverse events among different treatment groups.

Adverse Events	Treatment Groups (*n* = 184)				*p*-Value
-	AZA (*n* = 68)	DMF (*n* = 68)	TFL (*n* = 16)	IFN (*n* = 32)	
Total Adverse events number (%)	9 (13.24)	4 (5.97)	1 (6.25)	7 (21.88)	0.117
Infections	1 (1.47)	0	0	1 (3.13)	0.176
Leukopenia	7 (10.29)	0	0	1 (3.13)	**0.025**
Transaminitis	3 (4.41)	0	1 (6.25)	0	0.132
Flu-like symptoms	0	0	0	5 (15.63)	**0.0001**
Rash	0	3 (4.48)	0	1 (3.13)	0.284
Flushing	0	3 (4.48)	0	1 (3.13)	0.284
Injection site reactions	0	0	0	2 (7.14)	**0.035**

The *p*-values which are significant have been put in bold.

## Data Availability

Qualified researchers may request study protocol, statistical analysis, and patient-level data access. Patient data will be anonymized to protect the privacy of the participants.

## Abbreviations

AZA = Azathioprine, DMT = Disease-modifying therapy, DMF = Dimethyl fumarate, EDSS = Expanded Disability Status Scale, FLAIR sequence = Fluid Attenuated Inversion Recovery sequence, IFN beta-1a = interferon beta-1a, MAGNIMS = magnetic resonance imaging in multiple sclerosis, MS = Multiple Sclerosis, MRI = Magnetic Resonance Imaging, PPMS = Primary Progressive Multiple Sclerosis, RRMS = Relapsing-Remitting Multiple Sclerosis, RCT = Randomized control trial, SNR = Signal-to-noise ratio, SPMS = Secondary Progressive Multiple Sclerosis, TFL = Teriflunomide.

## Appendix A

Inclusion and Exclusion Criteria:

Inclusion Criteria:

1. Male or female patient (Age ≥ 18 to ≤55 years).
2. Patients with an established diagnosis of MS according to the 2010 McDonald Criteria
3. Patients with relapsing-remitting MS (RRMS) who were treatment-naive
4. Active disease as defined by Lublin 2014 [18], evidenced prior to Screening by:
  - At least 2 relapses in the last 24 months, or
  - At least 1 relapse in the last 12 months
  - A positive Gd+ MRI scan (brain and/or spine) in the last 12 months
  Relapses must have been assessed and documented by a physician in the patient files.
5. EDSS score between 0 and 5.5 (inclusive).
6. Female patients:
  - must be of non-childbearing potential, i.e., surgically sterilized (hysterectomy, bilateral salpingectomy, bilateral oophorectomy at least 6 weeks) or postmenopausal (where postmenopausal is defined as no menses for 12 months without an alternative medical cause), or
  - if of childbearing potential, must have a negative pregnancy test at (blood test) and (Day 1 blood or urine test). They must agree not to attempt to become pregnant, must not donate ova, and must use a highly effective contraceptive method (see below) together with a barrier method
  - highly effective forms of birth control are those with a failure rate of less than 1% per year and include:
    - oral, intravaginal, or transdermal combined (estrogen and progestogen containing) hormonal contraceptives associated with inhibition of ovulation.
    - oral, injectable, or implantable progestogen-only hormonal contraceptives associated with inhibition of ovulation.
    - intrauterine device or intrauterine hormone-releasing system.
    - bilateral tubal occlusion.
    - vasectomized partner (i.e., the patient’s male partner underwent effective surgical sterilization before the female patient entered the clinical study and is the sole sexual partner of the female patient during the clinical study).
    - sexual abstinence (acceptable only if it is the patient’s usual form of birth control/lifestyle choice; periodic abstinence [e.g., calendar, ovulation, symptothermal, postovulation methods] and withdrawal are not acceptable methods of contraception).
  - Barrier methods of contraception include:
    - condom.
    - occlusive cap (diaphragm or cervical/vault caps) with spermicidal gel/film/cream/suppository. Patient’s usual form of birth control/lifestyle choice), or
7. Willingness and ability to comply with the protocol.
8. Written informed consent

Exclusion Criteria:

MS-related exclusion criteria:

1. Patients with non-active secondary progressive MS and primary progressive MS.
2. Any disease other than MS that may better explain the signs and symptoms, including history of complete transverse myelitis.
3. Clinical signs or presence of laboratory findings suggestive of neuromyelitis optica (NMO) spectrum disorders or myelin oligodendrocyte glycoprotein (MOG)-IgG-associated encephalomyelitis.
4. Any MRI finding which puts in question the MS diagnosis, including but not limited to a longitudinally extensive spinal cord lesion.
5. History of malignancy of any organ system (other than localized basal cell carcinoma of the skin or adequately treated cervical cancer), treated or untreated, within the past 5 years, regardless of whether there is evidence of complete remission at the current time.
6. Any active and uncontrolled coexisting autoimmune disease other than MS (except for type 1 diabetes mellitus and inflammatory bowel disease).

## Appendix B

2010 Revised McDonald Diagnostic Criteria for MS:

Diagnosis of MS requires the elimination of more likely diagnoses and demonstration of dissemination of lesions in space and time

CLINICAL (ATTACKS)

LESIONS ADDITIONAL CRITERIA TO MAKE DX

2 or more Objective clinical evidence of 2 or more lesions or

Objective clinical evidence of 1 lesion with reasonable historical evidence of a prior attack

None. Clinical evidence alone will suffice; additional evidence is desirable but must be consistent with MS 2 or more Objective clinical evidence of 1 lesion

Dissemination in space, demonstrated by 1T2 lesion in at least two MS typical CNS regions

(Periventricular, juxtacortical, infratentorial, spinal cord); OR Await further clinical attack implicating a different CNS s

1 Objective clinical evidence of 2 or more lesions

Dissemination in time, demonstrated by

Simultaneous asymptomatic contrast-enhancing and non-enhancing lesions at any time; OR A new T2 and/or contrast-enhancing lesions(s) on follow-up MRI, irrespective of its timing; OR

Await a second clinical attack

1 Objective clinical evidence of 1 lesion

Dissemination in space, demonstrated by

1 T2 lesion in at least two MS typical CNS regions (periventricular, juxtacortical, infratentorial, spinal cord);

OR Await further clinical attack implicating a different CNS site AND

Dissemination in time, demonstrated by Simultaneous asymptomatic contrast-enhancing and

Non-enhancing lesions at any time; OR A new T2 and/or contrast-enhancing lesions(s) on follow-up MIR, irrespective of its timing; OR Await a second clinical attack 0 (progression from onset)

One year of disease progression (retrospective or prospective) AND at least 2 out of 3 criteria: Dissemination in space in the brain based on 1 T2lesion in periventricular, juxtacortical, or infratentorial regions; Dissemination in space in the spinal cord based on 2 T2 lesions; OR Positive CSF

Further Information on Diagnosing MS

What Is An Attack? Neurological disturbance of kind seen in MS Subjective report or objective observation At least 24 h duration in the absence of fever or infection Excludes pseudo attacks, single paroxysmal symptoms (multiple episodes of

Paroxysmal symptoms occurring over 24 h or more are acceptable as

Evidence) some historical events with symptoms and patterns typical for MS can provide reasonable evidence of

Previous demyelinating event(s), even in the absence of objective findings

Determining Time between Attacks 30 days between the onset of Event 1 and the onset of Event 2

What Provides Evidence for Dissemination in Space? 2

≥1 T2 lesion in at least two out of four areas of the CNS: periventricular, juxtacortical, infratentorial, or spinal cord. Gadolinium enhancement of lesions is not required for DIS. If a subject has a brainstem or spinal cord syndrome, the symptomatic lesions are excluded and do not contribute to the lesion count

What Provides MRI Evidence of Dissemination in Time? A new T2 and/or gadolinium-enhancing lesion(s) on follow-up MRI, with reference to a baseline, can, irrespective of the timing of the baseline MRI OR Simultaneous presence of asymptomatic gadolinium-enhancing and non-enhancing lesions at any time

What is Positive CSF?

Oligoclonal IgG bands in CSF (and not serum) or elevated IgG index
